# Opening of the Atlanta Polyclinic

**Published:** 1893-04

**Authors:** 


### OPENING OF THE ATLANTA POLYCLINIC.

The Atlanta Polyclinic was formally opened with appropriate exercises on March 22d. The President, Dr. F. W. McRae, gave the origin and history of the movement and described the object and scope of the institution. It was established to meet an indication, not to oppose any existing institution. It has been receiving outdoor patients for a month and the number now treated daily is very large. The Polyclinic was founded by the medical men now associated with it, and will be so maintained. The President then introduced Mr. Goodwin, the mayor of the city, who came to give the new enterprise his official good will. In a neat speech he pledged his official encouragement and co-operation to this good work. The President of the Atlanta Charitable Association, Dr. J. D. Turner, next delivered an excellent address full of sympathy and charity for the sick poor, the class of people that were to be benefited by the free dispensary of the Polyclinic. As a representative of the Charitable Association he would take pleasure in giving the Polyclinic his cordial support. Mr. Richardson, of the *Evening Journal*, also made an eloquent speech in which he paid a beautiful tribute to Atlanta's charitable spirit.

The Polyclinic, now fully organized, gives every promise of a successful career. The building is admirably adapted for the purpose, both in location and arrangement, and the clinical material has exceeded expectations. Its officers and teachers are thoroughly in earnest, and THE JOURNAL extends to them its hearty good wishes.

